# Genetic model

**DOI:** 10.1111/jcmm.12751

**Published:** 2016-01-14

**Authors:** Feifei Zhao, Manshu Song, Youxin Wang, Wei Wang

**Affiliations:** ^1^ School of Public Health Municipal Key Laboratory of Clinical Epidemiology Capital Medical University Fengtai District Beijing China; ^2^ School of Medical Sciences Edith Cowan University Perth WA Australia

*Dear Editor:*

We would like to comment on the article ‘*KCNQ1* rs2237892 C→T gene polymorphism and type 2 diabetes mellitus in the Asian population: a meta‐analysis of 15,736 patients’ by Li Yan‐yan *et al*. on the definition of genetic models (*J Cell Mol Med* 2014; 18 (2): 274–82). Classically, there are three genotypic test models, *i.e*. dominant/recessive/additive for exploring the genotypic and phenotypic association studies. In Li *et al*.'s article, unfortunately, the authors gave a wrong description on the dominant and recessive models.

Up to date, there is often no concrete evidence of the genetic mode of inheritance in the studies of complex disease genes. Most studies test multiple genetic models to explore the biological rationale behind the preference of these genetic models. Dominance of one of the alleles can be assumed by treating the heterozygote and one of the homozygote genotypes as a single category. For example, if the alleles of the gene of interest are A and B in haploid, and A is the ‘increasing’ or ‘risk’ allele, *i.e*. the one causing an effect, the three genotype groups would then be AA, AB and BB. This dichotomization of the SNP genotypes can be done as follows:

- Dominant: ‘AA + AB’ *versus* ‘BB’,
- Recessive: ‘AA’ *versus* ‘AB + BB’,
- Additive: ‘AA’ *versus* ‘AB’ *versus* ‘BB’.

In Li *et al*.'s study, when dominance of the T allele is assumed, the dominant genetic model would be ‘TT+CT’ *versus* ‘CC’, not ‘CC’ *versus* ‘CT+TT’. This is consistent with the recessive model ‘TT’ *versus* ‘CC+CT’ referred in this article. As a result, the carriers of rs2237892‐T (TT+CT) have a decreased risk for T2DM (OR = 0.69; 95% CI: 0.64–0.74) and not an increase risk as reported (OR = 1.45; 95% CI: 1.286–1.634). Thus, if we refer to C, the dominant genetic model would be ‘CC+CT’ *versus* ‘TT’, with ‘CC’ *versus* ‘TT+CT’ as a recessive model. Accordingly the other relevant calculations in the Li *et al*.'s paper should be corrected as well.

